# Knowledge, attitude and practice of vaccinators and vaccine handlers on vaccine cold chain management in public health facilities, Ethiopia: Cross-sectional study

**DOI:** 10.1371/journal.pone.0247459

**Published:** 2021-02-25

**Authors:** Solomon Ahmed Mohammed, Birhanu Demeke Workneh, Mesfin Haile kahissay

**Affiliations:** Department of Pharmacy, College of Medicine and Health Science, Wollo University, Dessie, Ethiopia; Federal University of Sergipe, BRAZIL

## Abstract

**Background:**

Effective management of the vaccine cold chain system at all levels is one of the crucial factors for maintaining vaccine potency. Vaccines require more complex handling and storage requirements due to increased temperature sensitivity and complicated immunization schedules. This urges adequate knowledge, attitude, and practice. This study assessed the knowledge, attitude, and practice of vaccinators and vaccine handlers’ in public health facilities.

**Methodology:**

An institutional-based cross-sectional study design was used to assess the knowledge, attitude, and practice of 127 vaccinators and vaccine handlers in public health facilities of Oromia Special Zone, from September 1 to 30, 2019. Data were collected using self-administered questionnaires and a structured observation checklist. Descriptive and inferential statistics were made using the statistical package for social sciences version 20. Variables with a p-value <0.05 were taken as statistically significant.

**Result:**

The response rate was (96.94%). Sixty-eight (53.5%; 95% CI: 46.5%, 61.4%), 58 (45.7%; 95% CI: 37.8%, 53.5%) and 62 (48.8%: 95% CI; 41.7%, 56.7%) vaccinators and vaccine handlers had satisfactory knowledge, positive attitude and good practice respectively. Receiving training on cold chain management had a statistically significant association with the level of knowledge on cold chain management (AOR = 3.04, 95% CI: 1.04–8.88).

**Conclusions:**

More than half of vaccinators and vaccine handlers had satisfactory knowledge, while below half of vaccinators and vaccine handlers had a positive attitude and good practice. The determinants of knowledge in cold chain management were receiving training on cold chain management. Providing regular technical support and on the job training on vaccine cold chain management will improve the knowledge, attitude, and practice of vaccinators and vaccine handlers.

## Introduction

Vaccination is one of the most powerful and cost-effective of all health interventions [1]. Vaccines are expensive products that save millions of children’s lives each year [2]. The introduction of a new vaccine can have a significant impact on a country’s health system [3] and require new strategies and additional cold storage capacity [4].

Vaccines are sensitive biological products that can easily be destroyed if handled incorrectly [2]. Exposure to inappropriate conditions can affect the potency of refrigerated vaccine [5]. The loss of vaccine potency may also cause the vaccine to become more reactogenic. Vaccines require more complex handling and storage requirements due to increased temperature sensitivity and complicated immunization schedules. This urges adequate training and supervision [6].

In the United States of America, health professionals’ median knowledge on vaccine program score was 47.6% [7]. The knowledge of vaccination against Human Papillomavirus infection was low (27.9%) among physicians in Hong Kong [8]. In India, the overall knowledge regarding cold chain practices was satisfactory [9, 10]. A review of evidence in Europe showed gaps in knowledge and poor communication among healthcare workers [11]. Knowledge of health professionals on the cold chain was not as per required levels to support effective cold chain management in Mozambique [12]. A study conducted in Nigeria showed an inadequate implementation of vaccine management guidelines [13] and about 43.0% health workers in Nigeria had good knowledge of vaccine management, while 66.1% of health workers in Nigeria had good vaccine management practices [14]. Another study in the health care facilities of Southern Nigeria showed that knowledge on appropriate management of the cold chain in two districts was poor [15]. Also in other previous studies, 272 (64.0%) personnel in Nigeria were found to have poor knowledge [16] and 7 (28.3%) personnels in Cameroon did not know the correct vaccine storage temperature [17]. About 124 (67.8%) vaccine providers in South Ethiopia responded correctly to the recommended range of temperature for storage vaccine [18] and 84% of health workers in Nigeria had good knowledge of vaccine vial monitor (VVM) [19].

Cold chain management, training, supervision, a higher level of education, and year of service were significant determinants of the practice of vaccine cold chain management [14, 15, 20]. Besides, vaccine supply chain performance and logistics in the health facilities were sub-optimal [21]. The inefficient vaccine management systems, including poor stock management, poor quality of vaccine handling and storage, contribute to high wastage of vaccines [4].

Vaccine wastage could be expected in all programs and some level of wastage is unavoidable [22]. Due to the increasing vaccine costs, countries are looking more closely than before at vaccine wastage [3]. Effective management of the vaccine cold chain system at all levels is one of the crucial factors for maintaining vaccine potency [2], which narrows the gap between vaccinated and immunized [9]. It saves program costs, prevents high wastage rates and stock-outs, and improves the safety of immunizations [23]. Significant improvements can also be made in cold chain management, resulting in considerable savings in vaccine and children’s life [24]. Thus, this study assessed the knowledge, attitude, and practice of vaccinators and vaccine handlers in public health facilities in the Oromia Special Zone.

## Methodology

### Study area and period

This study was conducted in public health facilities in the Oromia Special Zone, Ethiopia, from September 1 to September 30, 2019. The Oromia Special Zone is one of the ten zones found in the Amhara regional state. The administrative zone had 2 town administrations and 5 Woredas. The special zone had 2 hospitals, 28 health centers, and 115 health posts serving a total population of 459,847.

### Study design

An institutional-based cross-sectional study design was used to assess the knowledge, attitude, and practice of vaccinators and vaccine handlers who engaged in vaccine cold chain management.

### Source population

All vaccinators and vaccine handlers who engaged in vaccine cold chain management.

### Sample population

All vaccinators and vaccine handlers who engaged in vaccine cold chain management and who gave their consent to participate.

### Sample size determination and sampling procedures

All vaccinators and vaccine handlers (127) who engaged in vaccine cold chain management activities of the 27 public health facilities were included. Four participants refused to participate. Moreover, one health center and both hospitals were excluded due to interruptions in rendering vaccination services at the time of the study.

### Variables

The outcome variables of the study were the knowledge, attitude, and practice of vaccinators and vaccine handlers. Socio-demographic characteristics, receiving training, and supportive supervisions were predictor variables.

### Data collection tools and procedures

Self-administered questionnaire was used to assess the knowledge and attitude of vaccinators and vaccine handlers adopted from the World Health Organization (WHO) [23, 25, 26]. Knowledge was assessed using a total of 21 questions and attitude toward vaccine cold chain management was scored using a total of 6 questions on a 5 point Likert scale. A structured observation checklist was adopted from the WHO to assess their practice [27]. The vaccine cold chain practice was assessed using a total of 25 questions under 9 domains. A value of 1 and 0 was assigned for correct and false responses respectively. The total knowledge, attitude, and practice score for each respondent and vaccine cold chain practice and infrastructure of health facilities were converted to percentages, and also mean score was calculated.

A pretest test was carried out in two health centers to test validity of the study tools and instruments in those facilities that were not part of the study area. The data were collected by three experienced pharmacists and the principal investigators coordinated the overall data collection. The reliability of the self-administered questionnaire was also checked by Cronbach’s alpha test. The structured observation checklist and self-administered questionnaire had an alpha value of 0.72 and 0.75 respectively. To assure the quality of the data, questionnaires were critically examined for completeness, accuracy, clarity, and consistency.

### Data management and analysis

The data were entered using EpiData version 4.6 and exported to Statistical Package for Social Sciences version 20 for further analysis. The multiple logistic regression was fitted, crude and adjusted odds ratios were calculated with 95% confidence intervals, and variables with p-value <0.05 were taken as statistically significant. The analysis of this study did not account for clustering by health facility and multiple comparisons.

#### Operational definitions

*Satisfactory knowledge*. Vaccinators and vaccine handlers who scored greater than the mean score.

*Positive attitude*. Vaccinators and vaccine handlers who scored greater than the mean score.

*Good practice*. Vaccinators and vaccine handlers who scored greater than the mean score.

### Ethical considerations

Ethical approval of research was done from the Ethics Review Committee of the College of Medicine and Health Science, Wollo University (CHMS/405/13/11) and Oromia Special Zone Health Department. Oral informed consent was also requested from the study participants and confidentiality was maintained.

## Results

One hundred twenty-seven (96.94%) vaccinators and vaccine handlers responded. There were 85(66.9%) male respondents. The respondents consisted of 20 to 42 years of age with a mean age and standard deviation (SD) of 25.95±3.81. Among the vaccinator and vaccine handlers included in the study, 68 (53.5%) were midwives. Concerning the level of education, 48 (37.8%) were diplomas, while 79 (62.2%) had a degree of work experience ranging from 4 months to 14 years with mean work experience and SD of 3.29±2.44 (Table 1).

**Table 1:** Socio-demographic characteristics of respondents in public health facilities, Ethiopia, 2019 (n = 127).

Sr. no	Socio-demographic profile		Number	Percentage
1	Sex	Male	85	66.9
		Female	42	33
2	Age	20–24	49	38.6
		25–29	57	44.9
		≥30	21	16.5
3	Work experience	<5	93	73.2
		≥5	34	26.8
4	Level of education	Diploma	79	62.2
		Degree	48	37.8
5	Designation	Midwifery	68	53.5
		Health officer	9	7.1
		Nurse	50	39.4
6	Salary (Ethiopian birr)	1651–3200	70	55.1
		3201	43	33.9
		>5251	14	11
7	Received training on cold chain management	Yes	31	24.4
		No	96	75.6
8	Time of last training	Less than 6 month	14	45.2
		6–12 months ago	11	35.5
		Greater than a year ago	6	19.3
9	Receive supervision	Yes	35	27.6
		No	92	72.4
10	Time of last supervisory visits	Less than 6 month	15	42.9
		6–12 months ago	12	34.3
		Greater than a year ago	8	22.8

The correct placement of the thermometer was answered by 19 (15%). Only 23 (18.1%) vaccinators and vaccine handlers knew about to estimate vaccines need. Forty-nine (38.6%) vaccinators and vaccine handlers knew the three vaccines that required a shake test and the reason for the application of the shake test was correctly mentioned by 72 (56.7%). Proper compartment for placement of Oral Polio Vaccine (OPV), Bacillus-Calmette-Guerin (BCG), and Measles and Injectable Polio Vaccine (IPV), Tetanus Toxoid (TT), Penta-valent Vaccine (PV), Pneumococcal Conjugate Vaccine (PCV) and Rota vaccines in the top opening ice-lined refrigerators were correctly described by 44 (34.6%) and 33 (26%) of vaccinators and vaccine handlers, respectively (Table 2). Sixty-eight (53.5%; 95% CI; 46.5%, 61.4%) had satisfactory knowledge.

**Table 2:** Knowledge of respondents on key cold chain management areas in public health facilities, Ethiopia, 2019 (n = 127).

Sr.no	Key cold chain management areas	Frequency	Percentage
1	Placement of thermometer in ice lined refrigerators	19	15
2	Forecasting vaccines need	23	18.1
3	Placement of IPV, TT, PV, PCV, and Rota in top-opening ice lined refrigerators	33	26
4	Placement of BCG, OPV, and Measles in top-opening ice lined refrigerators	44	34.6
5	Vaccines needing shake test	49	38.6
6	Use of vaccine vial monitor	63	49.6
7	Most freeze sensitive vaccines	67	52.8
8	Most heat sensitive vaccines	67	52.8
9	Reason for vaccine shake test	72	56.7
10	Most light sensitive vaccines	72	56.7
11	Vaccine inventory management	74	58.3
12	Vaccines management in case of refrigerator/power break	77	60.6
13	Vaccines in the multi-dose vial policy	82	64.6
14	Recommended storage temperature range of diluents	89	70.1
15	Length of time to keep vaccines in vaccine carrier	91	71.7
16	Vaccines to be discarded within 6 hours after being opened	99	78
17	Vaccines requiring conditioned icepacks during transportation	100	78.7
18	Frequency of recording of vaccine refrigerator temperature	106	83.5
19	Recommended storage temperature range of vaccines	123	96.9

Forty-one (32.3%), 33 (26%), 40 (31.5%), and 24 (18.9%) vaccinators and vaccine handlers perceived that placing food and drinks with vaccines, opening refrigerators greater than 3 times a day, usage of reconstituted vaccines after 6 hours and usage of vaccine after expiration was acceptable (Table 3). Fifty-eight (45.7% 95% CI: 37.8%, 53.5%) and 62 (48.8%; 95% CI: 41.7%, 56.7%) had positive attitudes and good practice, respectively.

**Table 3:** Attitude of respondents on cold chain management in public health facilities, Ethiopia, 2019 (n = 127).

Sr. no	Description	Strongly disagree n(%)	Disagree n(%)	Neutral n(%)	Agree n(%)	Strongly agree n(%)	Mean± SD
1	Placing food and drinks with vaccines in the refrigerator affect vaccine potency	27(21.3)	14(11)	10(7.9)	30(23.6)	46(36.2)	3.43±1.57
2	An “open when needed label” be placed on the door of every vaccine refrigerator	12(9.4)	27(21.3)	16(12.6)	40(31.5)	32(25.2)	3.42±1.32
3	Vaccine refrigerators should be opened < 2 times a day	10(7.9)	23(18.1)	18(14.2)	38(29.9)	38(29.9)	3.56±1.3
4	Reconstituted vaccines should be used before 6 hour	17(13.4)	23(18.1)	6(4.7)	34(26.8)	47(37)	3.56±1.47
5	Vaccines should be used before expiration	15(11.8)	9(7.1)	4(3.1)	15(11.8)	84(66.2)	4.13±1.42
Average		16(12.8)	19(15.1)	11(8.5)	31(24.7)	49(38.9)	3.62±1.42

Only receiving training in cold chain management had a statistically significant association with the level of knowledge on cold chain management. The adjusted model indicated that health professionals who received training in cold chain management were about 3.04 times more likely to have satisfactory knowledge on cold chain management compared to those who did not receive training (Adjusted OR = 3.04, 95% CI: 1.04–8.88) (Table 4).

**Table 4:** Logistic regression of knowledge of respondents on cold chain management in public health facilities, Ethiopia, 2019 (n = 127).

Sr. no	Descriptions	Level of knowledge		Crude OR (95% CI)	Adjusted OR (95% CI)
		Non satisfactory	Satisfactory		
1	Work experience				
	<5	46(49.5%)	47 (50.5%)	0.63(0.28–1.41)	0.79(0.33–1.89)
	≥5	13(38.2%)	21(61.8%)	1.0	1.0
2	Level of education				
	Diploma	38(48.1%)	41(51.9%)	1.19(0.57–2.45)	0.95(0.41–2.16)
	Degree	21(43.8%)	27(56.2%)	1.0	1.0
3	Profession				
	Health officer	4(44.4%)	5(55.6%)	0.83(0.19–3.48)	1.12(0.22–5.55)
	Midwifery	20(40%)	30(60%)	0.62(0.3–1.31)	1.02(0.44–2.35)
	Nurse	35(51.4%)	33(48.6%)	1.0	1.0
4	Received training				
	Yes	7(22.6%)	24(77.4%)	0.24(0.09–0.62)	3.04(1.04–8.88)*
	No	52(54.2%)	44(45.8%)	1.0	1.0
5	Receive supervision				
	Yes	10(28.6%)	25(71.4%)	0.35(0.15–0.81)	1.77(0.68–4.57)
	No	49(53.3%)	43(46.7%)	1.0	1.0

*P-value < 0.05.

None of the variables were found to have a statistically significant association with the level of attitude on cold chain management. The age and salary of vaccinators and vaccine handlers had a statistically significant association with the level of cold chain management practice in the crude model. Vaccinators and vaccine handlers who earned a salary of 1651–3200 and 3201–5250 birr were about 2.54 (Adjusted OR = 2.54, 95% CI: 0.77–8.38) and1.42 times (Adjusted OR = 1.42, 95% CI: 0.40–4.96) more likely to have poor cold chain management practice compared to those who earn greater than 5251 Ethiopian birrs (Table 5).

**Table 5:** Logistic regression of practice of respondents on cold chain management in public health facilities, Ethiopia, 2019 (n = 127).

Sr. no	Descriptions	Cold chain practice		Crude OR (95% CI)	Adjusted OR (95% CI)
		Good	Poor		
**1**	Age				
	20–24	18(36.73%)	31(63.27%)	2.29(0.81–6.50)*	1.29(0.28–5.81)
	25–29	32(56.14%)	25(43.86%)	1.04(0.37–2.86)*	0.69(0.18–2.52)
	>30	12(57.14%)	9(42.86%)	1.0	1.0
**2**	Work experience				
	<5	42(45.16%)	51(54.84%)	0.57(0.26–1.27)	1.21(0.39–3.72)
	≥5	20(58.82%)	14(41.18%)	1.0	1.0
**3**	Salary				
	1651–3200	29(41.43%)	41(58.87%)	2.54(0.77–8.38)*	1.98(0.54–7.18)
	3201–5250	24(55.81%)	19(44.19%)	1.42(0.40–4.96)*	1.44(0.40–5.19)
	>5251	9(64.29%)	5(35.71%)	1.0	1.0
4	Received training				
	Yes	19(61.29%)	12(38.71%)	1.95(0.58–4.45)	0.53(0.22–1.26)
	No	43(62.29%)	53(37.71%)	1.0	1.0

*P-value < 0.05.

## Discussion

Only 24.4% of vaccinators and vaccine handlers received cold chain management-related training while just 27.6% got supervised on cold chain management. This was comparable with previous studies done in the past where the proportion of respondents not trained were 66.8% in central Ethiopia [28] and 78.1% in the Bale zone [18]. This was much lower than studies in Nigeria (65.0%) [14] and Brazil (91.3%) [29]. This might be attributed to a lack of management ownership, loss of attention on the cold chain, health professional attrition, and financial constraints.

The recommended range of temperature for vaccines was correctly responded by 96.9% in this study. This was comparable with a study done in central Ethiopia, where 78.4% responded correctly [20] but 1.42 (67.8%) and 1.35 (71.1%) times higher than those in Bale [18] and Guragie zone [28] respectively. Another study in Nigeria [30] and Mozambique [12] also reported that nearly half of the respondents (52.1%) knew the optimal vaccine storage temperature.

Regarding the frequency of temperature recording, 83.5% was correctly described in the present study. This was in line with findings in the Guragie zone, where 83.6% of health workers knew the frequency of temperature recordings [28], but 1.44 fold higher (57.9%) than respondents in the Bale zone [18] and central Ethiopia [20]. On the other hand, the correct placement of the thermometer was mentioned by15% in the current study and the result was much lower than Brazil (69%) [29].

In the present study, 38.6% of vaccinators and vaccine handlers knew vaccines that required a shake test and the finding was 1.9 fold lower than the study in Addis Abeba (73.8%) [31]. The reason for the application was correctly mentioned by 56.7%. This finding was much higher than a study conducted in the Bale zone (36.2%) [18], central Ethiopia (36.2%) [20], and Nigeria (51.6%) [30].

The types of vaccines that were most sensitive to freeze, heat, and light were correctly identified by 52.9%, 52.9%, and 55% of respondents, respectively. It was much better than study in Ethiopia [20, 31]. The knowledge of freeze sensitive vaccines was lower than Nigeria (77.9%) [16], Bale zone (60.1%) [18], and Addis Abeba (65.4%) [31], while the knowledge on heat and light sensitivity was lower (89.9%) and higher (42.6%) than Bale zone respondents, respectively [18]. Half (49.6%) of vaccinators and vaccine handlers knew the importance of vaccine vial monitor and this finding was higher than the study done in Addis Abeba (16.8%) [31] and Nigeria (45.3%) [30], but lower than the study conducted in the Bale zone (56.8%) [18].

Vaccine management in the case refrigerator out of function was known by 60.6% and the finding was comparable with central Ethiopia (63.8%) [20] but higher than the Bale zone (44.3%) [18]. When there is an equipment failure, larger quantities of vaccine can be destroyed [2]. During a malfunction, immediate steps should be taken to bring it back to working order [32]. To avoid the breakdown of preventive and curative maintenance systems to storage buildings and vaccine cold chains, equipment should be done [33] and there must also be a recording and reporting system of breakdowns [2].

One-third (34.6%) vaccinators and vaccine handlers described proper compartment for placement of OPV, BCG, and measles in this study and much less than a study conducted in Bale zone (67.2%) [18] and central Ethiopia (71.6%) [20]. This finding was in line with a study done in Cameroon, in which 37.7% of health personnel did not know the right place to store the measles vaccine in the refrigerator [17]. In Nigeria, 41.9% and 64.2% had a bad practice of storing freeze and heat-sensitive vaccines, respectively [15]. This might be due to insufficient numbers and inadequately trained staff to handle vaccines [34] or staff turnover. Temperatures may fluctuate in different compartments within the refrigerator, and vaccines should only be stored in certain areas [35].

In this study, 64.6% of vaccinators and vaccine handlers correctly listed vaccines under a multi-dose vial policy and the result was comparable with central Ethiopia (62.9%) [20]. Nearly one-third of respondents incorrectly interpreted the multi-dose vial policy [31]. This discrepancy in knowledge across studies might be attributed to participant’s qualification status, place of study, socio-cultural and demographic differences, and ways of assessing knowledge among studies.

The present study revealed that 53.5% of vaccinators and vaccine handlers had satisfactory knowledge. It was consistent with a study done in central Ethiopia and Bale zone, where 54.3% [20], Guragie zone (51.3%) [28], and 54.6% [18] had satisfactory knowledge. This finding was also better than studies in Nigeria [14–16, 36] and India [9, 10, 37], where knowledge of health personnel on cold chain management was poor, but in line with studies conducted in India [38, 39] and Malaysia [40], where knowledge on cold chain management was good. The discrepancy might be due to a difference in staff motivation, study participants’ qualifications, characteristics of the country, and the nature of the study.

In this study, 45.7% of vaccinators and vaccine handlers had a positive attitude towards cold chain management. Although the result was better than a study done in Nigeria, where 79.8% had a poor attitude towards cold chains [40], it is much less than the study conducted in Nigeria (93.2%) [16]. This wide discrepancy among vaccinators and vaccine handlers might be due to geographical and sociocultural differences among studies.

Sixty-two (48.8%) vaccinators and vaccine handlers had a good practice of cold chain management in this study. The finding was lower (73%) than the practice of cold chain management in Nigeria [14, 15]. Dairo and Osizimete portrayed that health professionals who had good knowledge and received formal training on vaccine cold chain management were more likely to have good practices [14, 39]. Further increasing the level of education will also improve the practice of cold chain management by 5.2 [15]. Thus, regular training and effective monitoring are recommended to ensure standard immunization [41, 42].

The determinants of knowledge on cold chain management were receiving training. In this study, professionals who received training in cold chain management were about 3.04 times more likely to have satisfactory knowledge of cold chain management compared to those who didn’t receive it. In Egypt, the total knowledge score was higher among healthcare providers who received training courses [42]. Another study in Nigeria showed that training was the determinant of cold chain management knowledge [29]. Moreover, healthcare workers who had sufficient training had better knowledge than healthcare workers who had no training [16, 43].

Despite socio-demographic characteristics of vaccinators and vaccine handlers were not found significant, professional qualification and year of service of health professionals working in the immunization program in Ethiopia [20] nurses having a bachelor degree in Egypt [42] showed a statistically significant association with knowledge of cold chain management. Inappropriate knowledge was also observed in those with longer formed (17.4 years) and a longer duration of vaccine units (7 years) [29].

In this study, attitude on vaccine cold chain management was not significantly associated with any of the variables. However, the level of education (P = 0.005) and knowledge of cold chain management (P < 0.001) were reported as determinants of attitude [16]. The age and salary of vaccinators and vaccine handlers had a statistically significant association with the level of cold chain management practice in the crude model. According to a study conducted in Southern Nigeria, significant determinants of the practice of cold chain management were cold chain management training (p = 0.004), a national program on immunization supervision (p < 0.001), and a higher level of education (p < 0.001) [15]. A similar study in Oyo State, Nigeria showed that health professionals who had good knowledge of vaccine handling and storage (p < 0.001) and had received formal training on vaccine management (p< 0.001) were 10 and 5.3 times more likely to have good vaccine handling and storage practices [14]. The regular practice was observed among those who had more time (8.3 years) working in vaccines cold chain management [29].

This study revealed that there were gaps in knowledge, practices, and attitudes of vaccinators and vaccine handlers regarding the vaccine cold chain system. The vaccine requires complex handling and storage requirements [2, 37] and each year storage and handling errors result in the revaccination of many patients and significant financial loss due to wasted vaccines [5]. Thus, assessing the knowledge, attitude, and practices of vaccinators and vaccine handlers will empower efforts made to develop the human resources required to manage vaccines and improve the immunization supply chain. However, the cross-sectional nature of this study might make it harder to establish a temporal relationship.

## Conclusion

More than half of vaccinators and vaccine handlers had satisfactory knowledge, while below half of vaccinators and vaccine handlers had a positive attitude and good practice. Receiving training on cold chain management was the determinant of knowledge on cold chain management and increased the odds of having better knowledge. Providing regular technical support and on the job training on vaccine cold chain management will improve the knowledge, attitude, and practice of vaccinators and vaccine handlers.

## Abbreviations

BCG: Bacillus-Calmette-Guerin
IPV: Injectable Polio Vaccine
OPV: Oral Polio Vaccine
PCV: Pneumococcal Conjugate Vaccine
PV: Penta-valent Vaccine
SD: Standard deviation
TT: Tetanus Toxoid
VVM: Vaccine vial monitor
WHO: World Health Organization
